# Remitting seronegative symmetrical synovitis with pitting edema syndrome in maintenance hemodialysis

**DOI:** 10.1002/iju5.12217

**Published:** 2020-09-08

**Authors:** Toru Matsugasumi, Tsuneyuki Nakanouchi, Kazuya Mikami, Takumi Shiraishi, Masatoshi Kadoya, Seijiro Toriyama, Hidefumi Taniguchi, Atsuko Fujihara, Fumiya Hongo, Osamu Ukimura

**Affiliations:** ^1^ Department of Urology Kyoto Prefectural University of Medicine Kyoto Japan; ^2^ Division of Nephrology and Urology Japanese Red Cross Kyoto Daiichi Hospital Kyoto Japan; ^3^ Division of Rheumatology Japanese Red Cross Kyoto Daiichi Hospital Kyoto Japan; ^4^ Toriyama Clinic Kyoto Japan

**Keywords:** hemodialysis, joint swelling and pain, RS3PE syndrome

## Abstract

**Introduction:**

The remitting seronegative symmetrical synovitis with pitting edema syndrome primarily occurs in elderly individuals to represent symptoms of edema, pain, and joint swelling. It could be misdiagnosed in elderly maintenance hemodialysis patients, as hemodialysis patients often present with pain and joint swelling induced by hypervolemia, inflammation, amyloidosis, and/or chronic kidney disease. Here, we describe a maintenance hemodialysis patient with remitting seronegative symmetrical synovitis with pitting edema syndrome.

**Case presentation:**

A 71‐year‐old man on maintenance hemodialysis who complained of continuous pain and swelling of joints was diagnosed with remitting seronegative symmetrical synovitis with pitting edema syndrome on his clinical findings that revealed tenosynovitis at the joint without joint erosions and no elevation of anti‐cyclic citrullinated peptide antibody and rheumatoid factor. After administration of prednisolone, systemic edema, and pain improved in 2 days.

**Conclusion:**

Remitting seronegative symmetrical synovitis with pitting edema syndrome should be considered as a differential diagnosis in hemodialysis patients with edema and/or arthralgia.

**Abbreviations & Acronyms**
CCP: cyclic citrullinated peptide
CRP: C‐reactive protein
EORA: elderly onset of rheumatoid arthritis
Hb: hemoglobin
HLA: human leukocyte antigen
Ig: immunoglobulin
MMP‐3: matrix metalloproteinase‐3
NA: not available
PMR: polymyalgia rheumatica
RF: rheumatoid factor
RS3PE: remitting seronegative symmetrical synovitis with pitting edema
VEGF: vascular endothelial growth factor

**Keynote message**

We report a case of the RS3PE syndrome in a maintenance hemodialysis patient. RS3PE should be considered as a differential diagnosis in hemodialysis patients with edema and/or arthralgia.

## Introduction

McCarthy described the “RS3PE” syndrome in 1985.^1^ RS3PE syndrome is characterized by the sudden onset of bilateral symmetrical synovitis and seronegative inflammation with symmetrical pitting edema. RS3PE syndrome primarily occurs in elderly individuals and only rarely in hemodialysis patients. The symptoms of RS3PE syndrome could be misdiagnosed in elderly hemodialysis patients. To our knowledge, this is the third case of RS3PE syndrome reported in hemodialysis patients.^2^, ^3^

## Case presentation

A 71‐year‐old man was admitted to our hospital following 5 weeks of sudden onset of continuous pain and swelling of the shoulder, wrist, and knee joints, bilaterally. The patient had been treated on an out‐patient basis by an orthopedic surgeon for high fever and arthritic symptoms, which were not improved with antibiotics and acetaminophen. The patient had a past medical history of bilateral renal pelvic and bladder cancer and had undergone bilateral nephroureterectomies and total cystectomy at ages 64, 66, and 70 years, respectively; followed by undergoing maintenance hemodialysis for 5 years. Pathological findings in bilateral nephroureterectomy without chemotherapy revealed urothelial carcinoma, high grade and pT2. Remained bladder was removed by cystectomy due to mucosal epithelial cancer after induction of hemodialysis.

Physical examination in hospitalization revealed a prominent pitting edema of the distal limbs, and mild tenderness of the peripheral joints of the entire body, with considerable pain on flexion of each joint. The degree of edema and pain were not changed between the hemodialysis and nonhemodialysis state. On laboratory testing, leukocyte count and CRP levels were 7.68 × 10^9^/L and 14.3 mg/L respectively. Blood and urine cultures were negative. Antinuclear antibody, anti‐CCP antibody, and RF were negative. The MMP‐3 level was markedly increased to 677.9 ng/mL (Table 1).

**Table 1:** Result of laboratory data at admission

			Reference range
Hematology			
White blood cells	7680	/μL	4000–8000
Red blood cells	2.71	×10^6^/μL	4.27–5.70
Hb	7.8	g/dL	13.5–17.6
Hematocrit	24.4	%	39.8–51.8
Platelets	374	10^3^/μL	150–350
Blood chemistry			
Total protein	5.5	g/dL	6.7–8.3
Albumin	2.2	g/dL	4.0–5.0
Aspartate aminotransferase	9	IU/L	13–33
Alanine aminotransferase	6	IU/L	8–42
Blood urea nitrogen	57	mg/dL	8–22
Creatinine	9.96	mg/dL	0.65–1.07
Sodium	136	mmol/L	138–146
Potassium	5.1	mmol/L	3.6–4.9
Chloride	104	mmol/L	99–109
Calcium	9.2	mg/dL	8.4–10.1
Glucose	98	mg/dL	70–109
HbA1c	5.7	%	−5.6
Serological examination			
CRP	14.3	mg/dL	<0.30
Erythrocyte sedimentation rate	71	mm/h	2–10
Free‐triiodothyronine	1.0	pg/mL	2.5–5.0
Free‐thyroxine	0.79	ng/mL	0.8–1.7
Thyroid stimulating hormone	2.55	μIU/mL	0.34–4.0
RF	<5	U/mL	<15
Anti‐CCP antibody	<0.6	U/mL	<4.5
MMP‐3	677.9	ng/mL	35.2–123.8
Antinuclear acid antibody	<40		<40
Proteinase‐3‐anti‐neutrophil cytoplasmic antibodies	<1.0	IU/mL	<2
Myeloperoxidase‐anti‐neutrophil cytoplasmic antibodies	<1.0	IU/mL	<3.5
IgG	1183	mg/dL	870–1700
IgA	272	mg/dL	110–410
IgM	49	mg/dL	35–220
50% hemolytic unit of complement	66	mg/dL	32–58
Complement 3	131	mg/dL	65–135
Complement 4	31	mg/dL	13–35

**Fig. 1:**
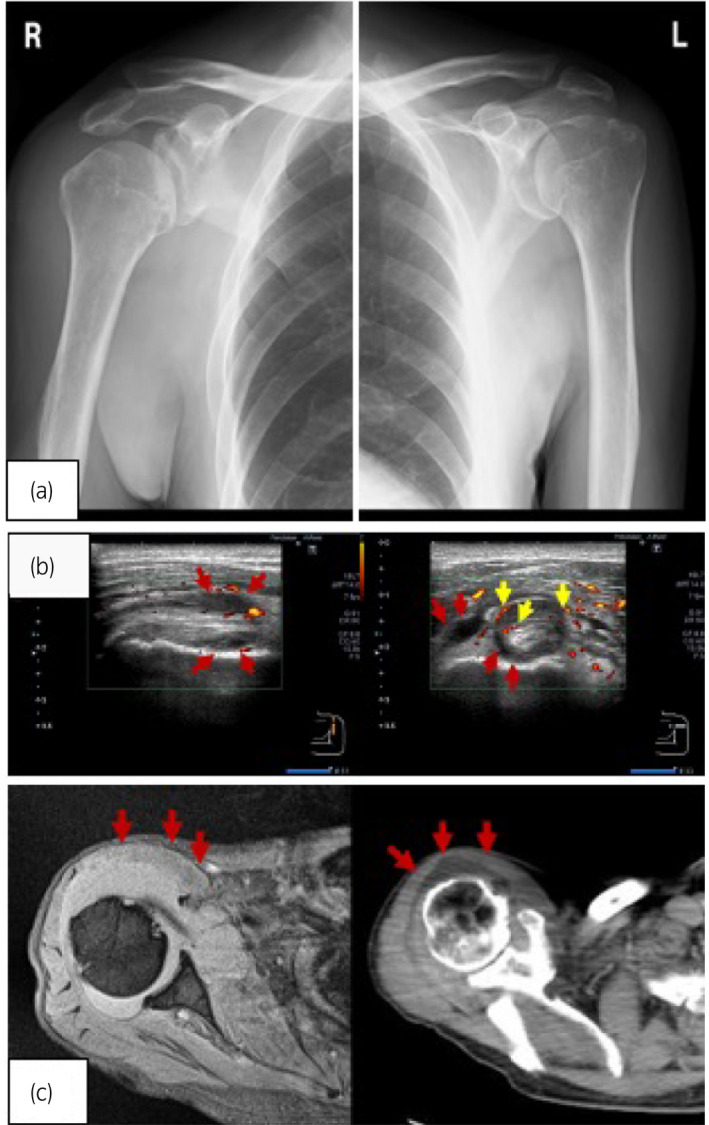
(a) Plain radiographs of the right and left shoulders showed no bone deformation, no marginal erosions, no localized osteopenia, or no fracture. (b) Doppler‐ultrasound revealed signs of tenosynovitis including synovial fluid (red arrows) of the biceps brachii and increased blood flow signal (yellow arrows). (c) Magnetic resonance imaging (left) and computed tomography (right) of the shoulder joint showed synovitis (arrows) without evidence of destructive images such as bone erosion.

On plain radiographs, there was no evidence of joint or bone deformation or erosion of the joint (Fig. 1a). Ultrasound examination (Fig. 1b) and magnetic resonance imaging (Fig. 1c) revealed tenosynovitis of the bilateral carpel joints and biceps brachii. Computed tomography revealed no evidence of malignant disease or liquid accumulation around the right shoulder joints (Fig. 1c).

A diagnosis of RS3PE syndrome was made, based on the symmetrical symptom of peripheral pitting edema without no serum reaction suggestive for PMR as following; acute onset of a prominent pitting edema of the symmetrical continuous pain and swelling of the joints, negative for anti‐CCP and RF whereas elevated CRP and MMP‐3 and no evidence of joint erosions on graphics. After oral prednisolone treatment (15 mg, once daily) was initiated, systemic edema, CRP level (decrease to 1.16 mg/dL) and pain improved over 2 days. Edema and joint pain were fully recovered, and the patient was discharged from hospital 9 days after the start of prednisolone treatment. No complications were identified during hospitalization, with no recurrence of the symptoms of peripheral edema and joint pain, with no recurrence for 6 months post‐treatment initiation. The dosage of prednisolone was gradually reduced, as maintenance hemodialysis was continued.

## Discussion

RS3PE syndrome is characterized by the following criteria: abrupt onset, marked pitting edema of the hands and/or feet, onset >60 years, good response to steroids, seronegative for RF and CCP, and absence of radiographic evidence of joint erosions. RS3PE is associated with VEGF, HLA‐A2, HLA‐B7, and HLA‐DRB1, as well as parvovirus and streptobacillus infections. Moreover, RS3PE can occur in association with paraneoplastic syndromes, such as leukemia and lung, breast, and bladder carcinoma.^1^, ^4^ RS3PE could also be induced by nivolumab.^5^ In a study of 331 patients, elevated VEGF levels was identified in five of 331 cases^4^ to suggest possible role for development of RS3PE. As VEGF causes hypervascularity and vascular permeability, an increase in VEGF may contribute to develop pitting edema in the dorsum of the hands and feet. The contribution of VEGF to develop RS3PE syndrome is supported by the improved edema after glucocorticoid treatment.^6^, ^7^ Serum MMP‐3 reportedly not only supports diagnosis of RS3PE syndrome, but also reflects the disease status and may be more sensitive than CRP.^8^ Although MMP‐3 was reported to be generated by some cancer, malignancy was not identified in this case. It is considered necessary to continue to observe the malignant tumors in follow‐up.

Differential diagnoses of RS3PE syndrome include PMR and elderly onset of EORA. Although RS3PE syndrome develops mainly in males involving the distal extremities with extensor tenosynovitis,^9^ PMR develops mainly in females, involving the proximal extremities with extensor tenosynovitis.^4^ Differentiation of RF‐negative EORA and PMR, with mild distal tenosynovitis is difficult. PMR and RS3PE are generally treated with low dose of glucocorticoids. However, EORA, associated with elevated CCP, is typically treated with methotrexate for rheumatoid arthritis.^7^ As such, there are no clear criteria for the diagnosis of RS3PE syndrome.

Only two cases of RS3PE syndrome were reported in hemodialysis patients at 9 and 23 years after the start of hemodialysis.^2^, ^3^ In both of these cases, there was no evidence of active inflammation and neoplasm. Although VEGF was not assayed in the first case,^2^ it was increased to 141 pg/mL in the second case^3^ (Table 2). In our case, the cardiothoracic ratio, before and after the onset of hemodialysis, was 52% and 55%, respectively, and weight gain was also within 3% of the dry weight, and the transient edema, as well as other symptoms, was not thought to be associated with hypervolemia. Symptoms could not be associated with any type of arthritis, with negative tests for the RF, anti‐CCP antibody, and anti‐nuclear antibody.

**Table 2:** Reported cases of dialysis patients with RS3PE syndrome

Reference	Age; gender	Hemodialysis duration (years)	Associated illness	Clinical feature	VEGF (reference range; <38 pg/mL)	Treatment
Yamada *et al*.^2^	75; male	23	NA	Bilateral wrist pain, dorsal edema of hands and feet.	NA	Prednisolone, 12 mg/day
Shindo *et al*.^3^	59; male	9	Diabetic nephropathy	Severe pitting edema and pain of metacarpophalangeal and proximal interphalangeal joints and wrists.	141 pg/mL	Methylprednisolone, 0.4 mg/kg body weight/day
Current case	71; male	5	NA	Sudden onset of continuous pain and swelling of the shoulder, wrist, and knee joints, bilaterally.	NA	Oral prednisolone, 15 mg/day

An excellent response with RS3PE syndrome to glucocorticoids treatment (0.3 mg/kg/day) is reported, with symptoms typically resolving within 1 week, regardless of hemodialysis status, with a relapse rate after treatment of 8.76%. The risk of relapse is higher for malignancy patients,^4^ who may be resistant to treatment. Among 10 patients with RS3PE syndrome and no evidence of malignancy, Russell reported that four patients developed a new onset of malignancy, which was statistically significant, and concluded that RS3PE syndrome is associated with a 20% higher rate of neoplasm.^10^ This rate is higher than the malignancy rate associated with PMR (0.44%) and EORA (0.56%).^7^ Patients with RS3PE syndrome should be monitored regarding the development of malignancy, especially when the patient with systemic symptoms such as fever, cachexia, and weight loss are observed. It should be noted that paraneoplastic RS3PE syndrome often has poor responsiveness for the steroid treatment.^11^

We propose that RS3PE syndrome should be considered as a differential diagnosis among elderly hemodialysis patients with edema, pain, swelling of joints, and distal extremities, rather than attributing these symptoms to hypervolemia, chronic inflammation, amyloidosis, and/or mineral and bone disorder associated with chronic kidney disease.

## Conclusion

In conclusion, although RS3PE syndrome is rare, RS3PE syndrome should be considered as a differential diagnosis in maintenance hemodialysis patients presenting with edema and/or arthralgia.

## Conflict of interest

The authors declare no conflict of interest.
